# Supplementary material for: OntoTrek: 3D visualization of application ontology class hierarchies
Source: PLoS One. 2023 Jun 2;18(6):e0286728. doi: 10.1371/journal.pone.0286728 (PMC10237395; doi:10.1371/journal.pone.0286728)
Supplement: Attachment — Submitted filename: Ontotrek Response to Reviewers.pdf [file pone.0286728.s001.pdf]

Dear Dr Chen, PLOS Editorial Office, and Reviewer,

We would like to thank you for your detailed and constructive review and suggestions. I (William Hsiao) apologize for the delay in sending back the review as a family member fell sick and passed away during the last few months as we were preparing for the responses. We have provided point-to-point reponses (in green) below for your consideration. We felt that updating the rendering engine to address some of the fundamental and valid concerns will require significant amount of work and fell outside of the scope of our current work. As a result, we tried our best to clarify the weaknesses and reinforce the utility of the tool as it current stands. We believe the community will still benefit from the compact visualization views that Ontotrek provides. Thank you.

Journal Requirements:

When submitting your revision, we need you to address these additional requirements.

1. Please ensure that your manuscript meets PLOS ONE's style requirements, including those for file naming. The PLOS ONE style templates can be found at

https://journals.plos.org/plosone/s/file?id=wjVg/PLOSOne_formatting_sample_main_body.pdf and

https://journals.plos.org/plosone/s/file?id=ba62/PLOSOne_formatting_sample_title_authors_affiliations.pdf

We have updated the style formats according to the template.

2. Thank you for stating the following financial disclosure:

"DD is funded by Genome Canada Project 286GET and the USDA NACA Contract 58-8040-8-014-F to WWLH. MHN is funded by CGS-M Scholarship."

Please state what role the funders took in the study. If the funders had no role, please state: "The funders had no role in study design, data collection and analysis, decision to publish, or preparation of the manuscript."

As requested "The funders had no role in study design, data collection and analysis, decision to publish, or preparation of the manuscript." has been added

If this statement is not correct you must amend it as needed.

Please include this amended Role of Funder statement in your cover letter; we will change the online submission form on your behalf.

3. PLOS requires an ORCID iD for the corresponding author in Editorial Manager on papers submitted after December 6th, 2016. Please ensure that you have an ORCID iD and that it is validated in Editorial Manager. To do this, go to 'Update my Information' (in the upper left-hand corner of the main menu), and click on the Fetch/Validate link next to the ORCID field. This will take you to the ORCID site and allow you to create a new iD or authenticate a pre-existing iD in Editorial Manager. Please see the following video for instructions on linking an ORCID iD to your Editorial Manager account: https://www.youtube.com/watch?v=_xcclfuvtxQ

We have added ORCID ID to the system

Reviewers' comments:

Reviewer's Responses to Questions

Comments to the Author

1. Is the manuscript technically sound, and do the data support the conclusions?

The manuscript must describe a technically sound piece of scientific research with data that supports the conclusions. Experiments must have been conducted rigorously, with appropriate controls, replication, and sample sizes. The conclusions must be drawn appropriately based on the data presented.

Reviewer #1: Partly

---

2. Has the statistical analysis been performed appropriately and rigorously?

Reviewer #1: N/A

---

3. Have the authors made all data underlying the findings in their manuscript fully available?

The PLOS Data policy requires authors to make all data underlying the findings described in their manuscript fully available without restriction, with rare exception (please refer to the Data Availability Statement in the manuscript PDF file). The data should be provided as part of the manuscript or its supporting information, or deposited to a public repository. For example, in addition to summary statistics, the data points behind means, medians and variance measures should be available. If there are restrictions on publicly sharing data—e.g. participant privacy or use of data from a third party—those must be specified.

Reviewer #1: Yes

---

4. Is the manuscript presented in an intelligible fashion and written in standard English?

PLOS ONE does not copyedit accepted manuscripts, so the language in submitted articles must be clear, correct, and unambiguous. Any typographical or grammatical errors should be corrected at revision, so please note any specific errors here.

Reviewer #1: Yes

---

5. Review Comments to the Author

Please use the space provided to explain your answers to the questions above. You may also include additional comments for the author, including concerns about dual publication, research ethics, or publication ethics. (Please upload your review as an attachment if it exceeds 20,000 characters)

Reviewer #1: The authors describe a 3-D visualization system for browsing the content of an ontology. One of the main motivations seems to be to be able to visualize which content from external terminologies is imported into the ontology and under which branches of the ontology it is found. In testing the interface is challenging to use. For any ontology with more than a tiny number of classes, it is hard to see the different nodes, and the UI zooms in and out confusingly. A positive aspect is that ontology content from different sources is colored differently, so that the source can be observed at a glance.

We recognize a problem exists in the 3d rotation / view, and have included a description of this in the main body text (line 300):

> *“Clicking on any node in the Ontotrek viewport causes the node to be highlighted in red, and also triggers a transition animation that moves the viewport camera perspective to an angled view close to and above the node. This built-in force-graph-3d feature is problematic and requires an alternative algorithm because the "rollercoaster" transition animation doesn't keep the viewport itself oriented vertically, so it is up to a user to try to realign the view after the transition has finished.”*

It is also mentioned via “important to eliminate changes in pitch and roll in the animation algorithm experienced when zooming into a node“ at end of discussion, line 351. Currently we use the built-in https://github.com/vasturiano/3d-force-graph/ navigation control system. However we envision replacing this with a navigation system which would focus on a given ontology or ontology part, and always maintain the viewing window vertically in line

with the ontology hierarchy (with possibly some tilt flexibility). We think this would bring better constancy to the viewport.

> I think there are three main aspects the authors could clarify or develop further that would help to make this system more usable:
>
> **1. Performance is a serious problem when browsing real world ontologies in the system.**

We have resolved one technical bug where trying to load successive ontologies from a pulldown menu selection or URL field was causing slower and slower rendering (this required a force-graph-3d destructor method between loads).

To some extent performance can be improved by disabling some rendering functionality as noted in line 291 beginning "There are a few Settings tab options that can improve rendering performance.", but ultimately "video-game tech" alternative approaches are required at some point for any rendering platform, which we now elaborate on in discussion section.

Generally, the force-graph-3d rendering engine is driven by as far as we know the fastest web-browser accessible technology, WebGL, if one is simply dealing with lines or "tube" edges, node spheres, and term labels.

> **2. The majority of usages of external content in OBO ontologies is via composition, rather than inheritance. For example an HPO phenotype is related to the affected anatomical structure. For this reason it would be valuable to traverse these kinds of links rather than just subClassOf.**

Allowing that our primary objective was to illustrate how OBO ontologies can be woven into an application ontology, we have revised the paper to briefly acknowledge caveats in displaying other kinds of relation or axiom, line 262:

> "As well, OntoTrek doesn't render other kinds of relations that axioms reference, nor will it show classes which only appear in axioms and not in the class hierarchy. This is primarily because OntoTrek's design objective is to focus on an informative display of the class hierarchy itself, and secondly because we have not embarked on the very careful work required to display other kinds of relations in a limited / local way that does not overwhelm the visual interface."

Some ontologies like UBERON have significant parthood connections to extend up to high-level entities, but for many ontologies, one would have to decide how to display all the "molecules" – where parts- say a wheel, nut, bolt, etc, is shared among many entities as parts.

3. The main issue that I think should either receive more development, or be addressed more clearly in the paper, is what purpose does the third dimension serve? It seems like many of their ideas (e.g., clearly denoting external content, showing the branching pattern from superclass to subclass) could be supported by a (possibly more scalable) two dimensional system. If one dimension denotes more-general-to-more-specific, and the second dimension separates peer terms from each other, what does change in the third dimension indicate?

In the paper we have added a few more points to our critique of the 2D display, line 241:

> “However, such a visualizer still struggles to represent large ontologies in a clean manner, often containing illegible labels and crowded layouts with criss-crossed edges when zoomed-out, and when zoomed-in, the challenge of tracing edges that journey out of viewport scope. Here "viewport" refers to a software application window where the interactive ontology is rendered.”

This is evident in slides 5,6,7,8,9 provided in a presentation ((screenshots attached; full presentation linked below) which are 2D snapshots from proftégé or other apps of ontology content visualization:
https://docs.google.com/presentation/d/1LheYra-39xgiV2JLYmYhhhddHPMI0ZDUcFLYPgmMa8Y

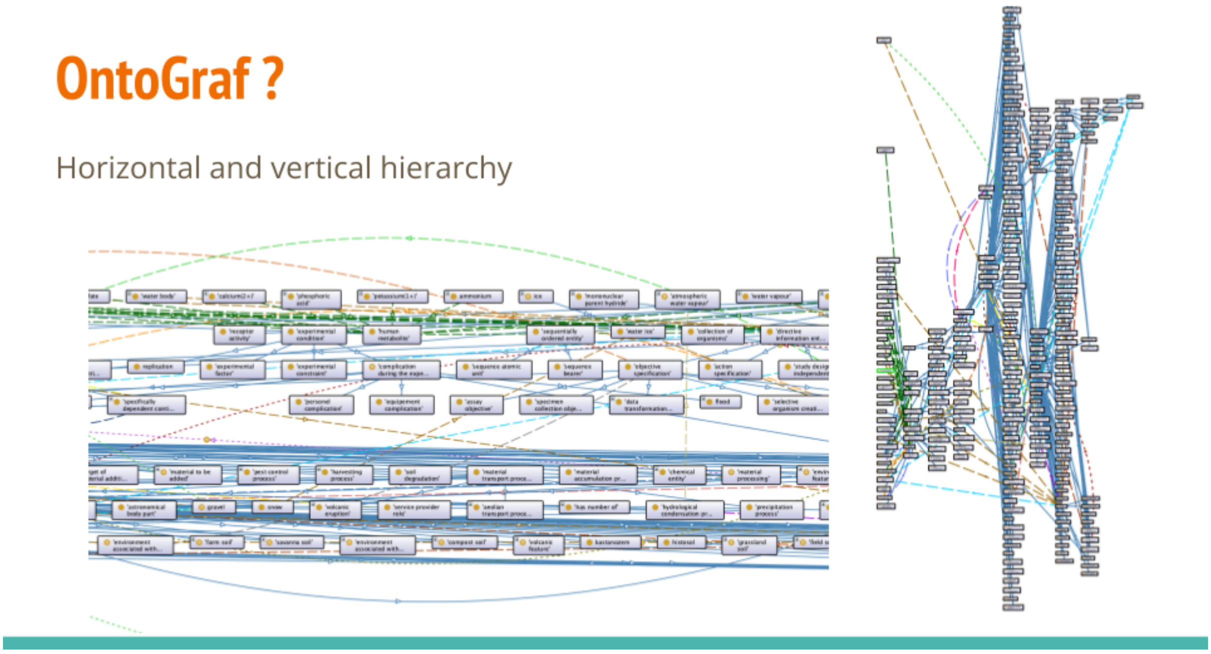

OntoGraph

Spring and Grid views

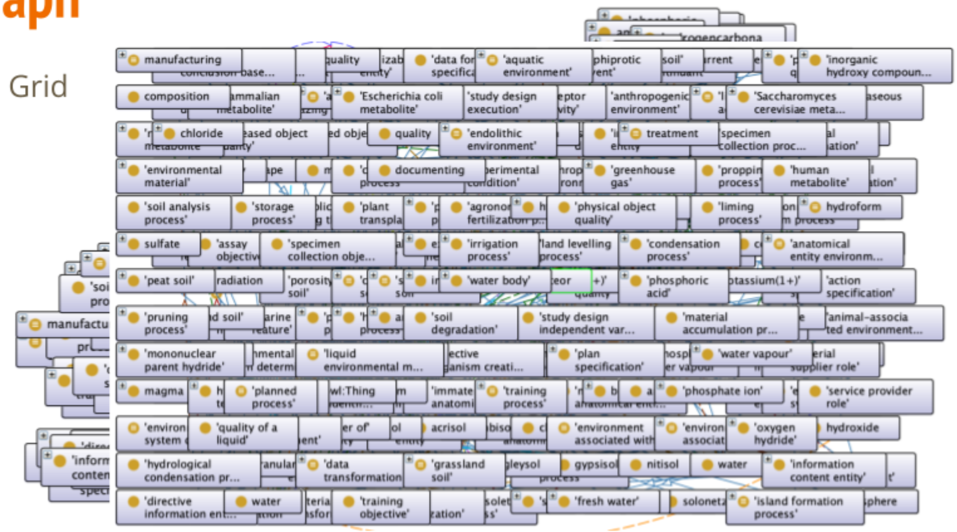

OWLViz

One screen of the AGRO ontology

Polyhierarchical

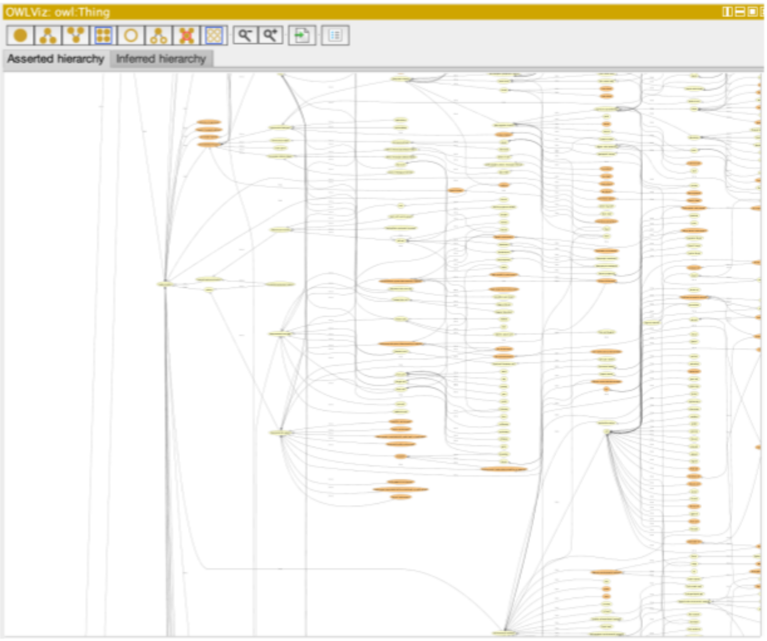

WebVowl (2014-)

Forte is showing many types of relation in a force-directed graph

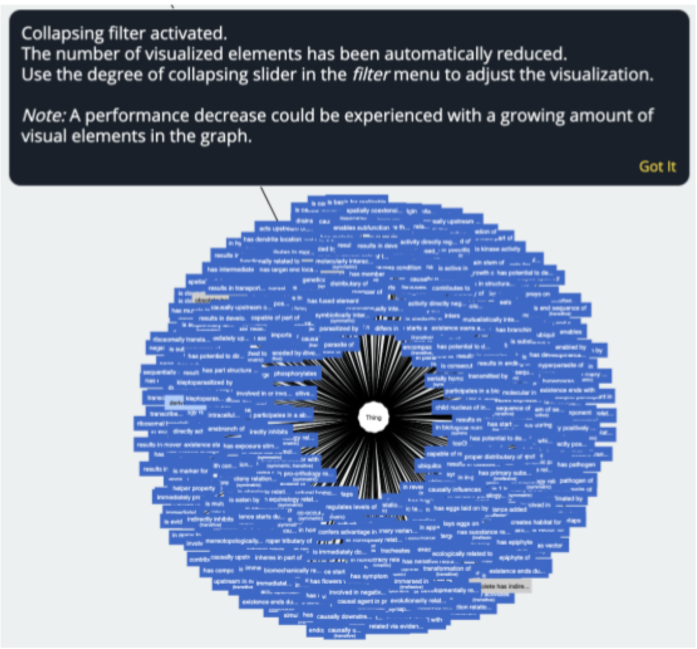

http://vowl.visualdataweb.org/webvowl.html

WebVowl

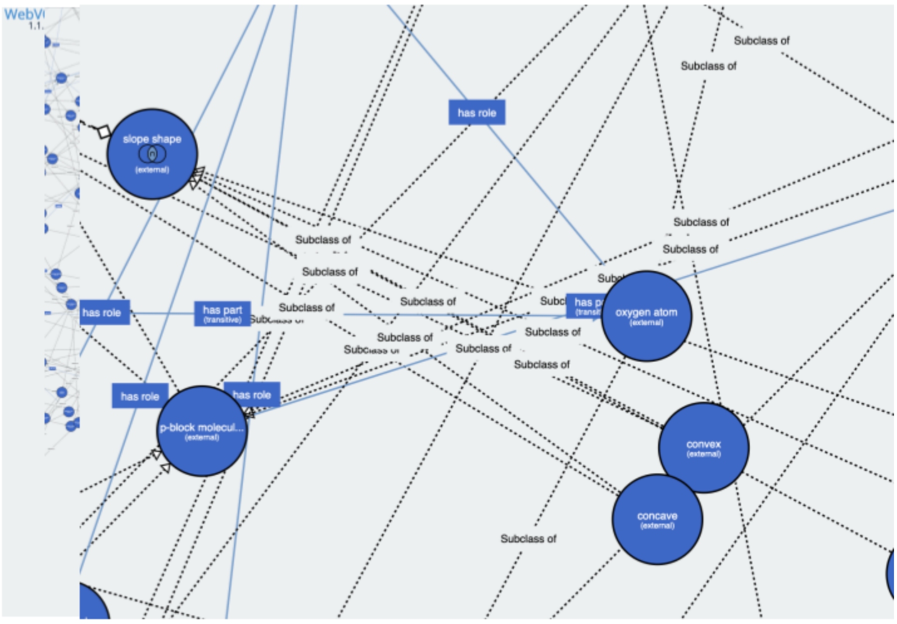

The Ontotrek system actually does have a hybrid 2D and 3D view, illustrating benefits of both combined, which now is mentioned in the text, line 280.

> “OntoTrek actually has a hybrid 3D and 2D view, available by toggling the "\textbf{Underlying ontology branches rendered as vertical slice(s)}" setting, which illustrates benefits of both combined by showing an upper level ontology in 3D, and underlying ontologies in vertical planes of 2D hierarchies. This option provides a fly-through view that separates out the labels which is quite effective with a medium sized ontology like AGRO or GenEpiO. The algorithm, like the general 3D one, currently doesn’t space out the lower level nodes and their labels very well, and so could be improved.”

We maintain that a 3D view, or 3D + 2D view, offers a more compressed way to show all the information. Our approach seeks in the 3D view to position information much like apartment addresses situated in a city of high-rises, rather than via a flat map view.

---

6. PLOS authors have the option to publish the peer review history of their article (what does this mean?). If published, this will include your full peer review and any attached files.

If you choose “no”, your identity will remain anonymous but your review may still be made public.

Do you want your identity to be public for this peer review? For information about this choice, including consent withdrawal, please see our Privacy Policy.

Reviewer #1: No
